# Supplementary material for: Structure-Property Relationships of 2D Ga/In Chalcogenides
Source: Nanomaterials (Basel). 2020 Nov 2;10(11):2188. doi: 10.3390/nano10112188 (PMC7693234; doi:10.3390/nano10112188)
Supplement: Supplementary file 1 [file nanomaterials-10-02188-s001.pdf]

nanomaterials

Supplement Materials: Investigation on structure-property relationships of 2D Ga/In chalcogenides

Pingping Jiang [1], Pascal Boulet [1,*] and Marie-Christine Record [2]

[1] Aix-Marseille Univ., CNRS, MADIREL, 13013 Marseille, France; pingping.jiang@etu.univ-amu.fr
[2] Aix-Marseille Univ., Université de Toulon, CNRS, IM2NP, 13013 Marseille, France; m-c.record@univ-amu.fr
* Correspondence: pascal.boulet@univ-amu.fr; Tel: +33-413-55-18-10

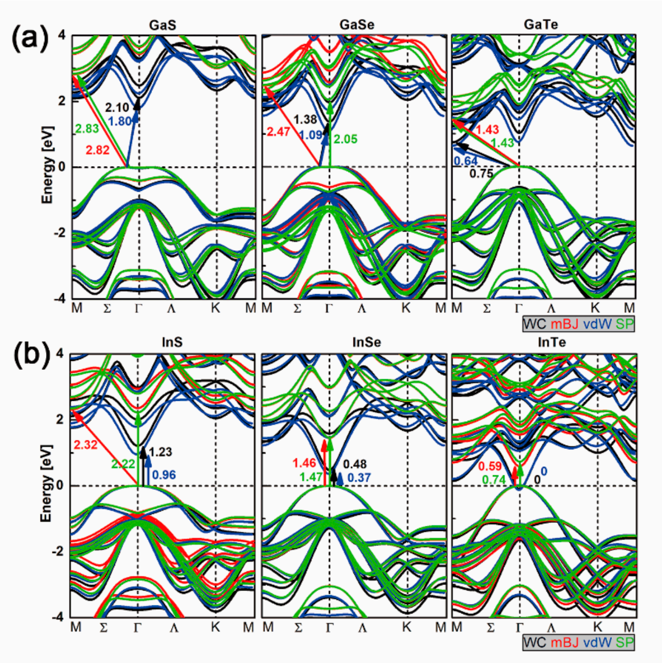

Figure S1. Band structures of bulk GaX **(a)** and InX **(b)** (X = S, Se, Te) calculated using WC-GGA with (green curves) and without polarization (black curves), mBJ (red curves) and optB88-vdW (blue curves) functionals.

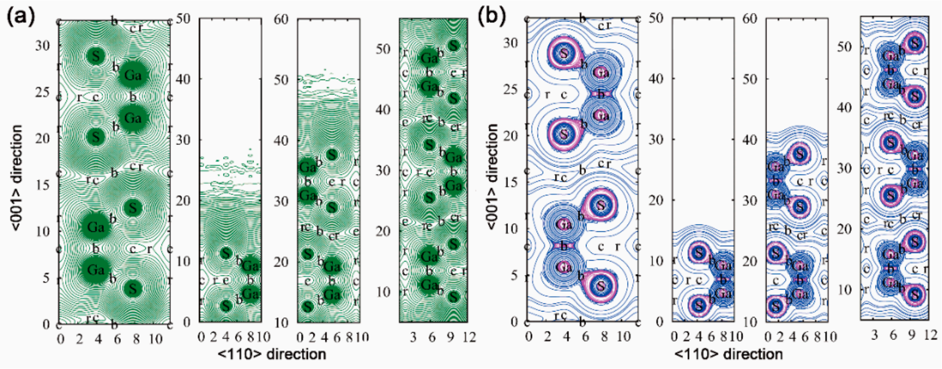

Figure S2. Electron density **(a)** and Laplacian **(b)** distributions of bulk, mono-, bi, and trilayered GaS in (−110) plane. Labels "b", "r" and "c" represent bond, ring and cage critical points at the zero-flux surface respectively.

nanomaterials

MDPI

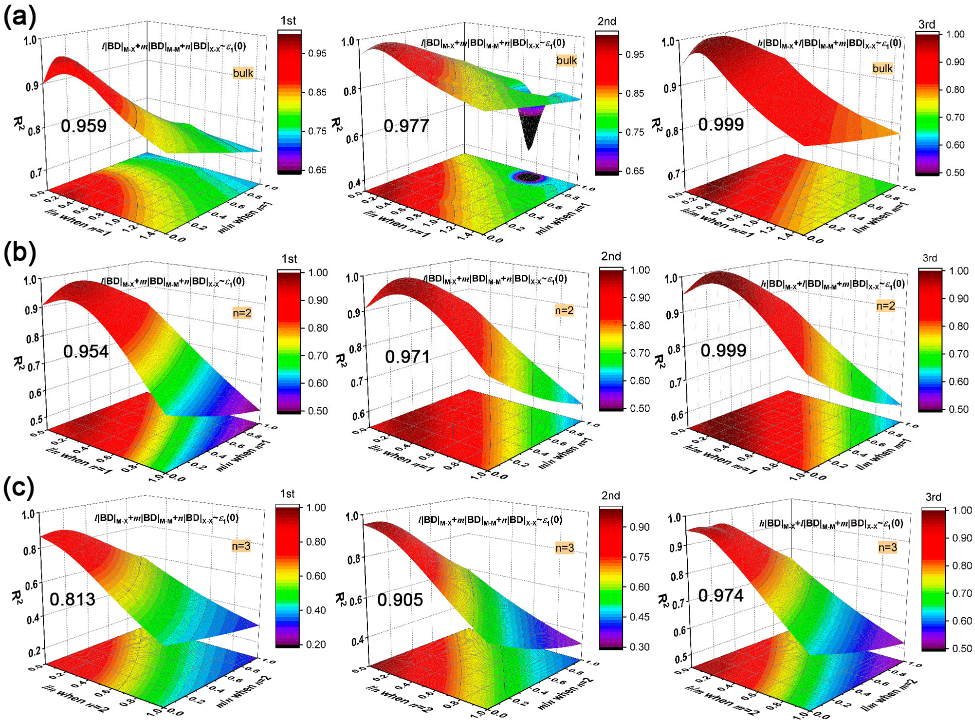

Figure S3. Polynomial fitting $h|\mathrm{BD}|_{\mathrm{M\text{–}X}} + l|\mathrm{BD}|_{\mathrm{M\text{–}M}} + m|\mathrm{BD}|_{\mathrm{X\text{–}X}}$ vs. $\varepsilon_1(0)$ as equation order goes from the first to second and to third: Coefficient of determination R^2 of bulk **(a)**, bilayer **(b)**, trilayer **(c)** by adjusting h/m and l/m ratios and their respective maximum R^2.

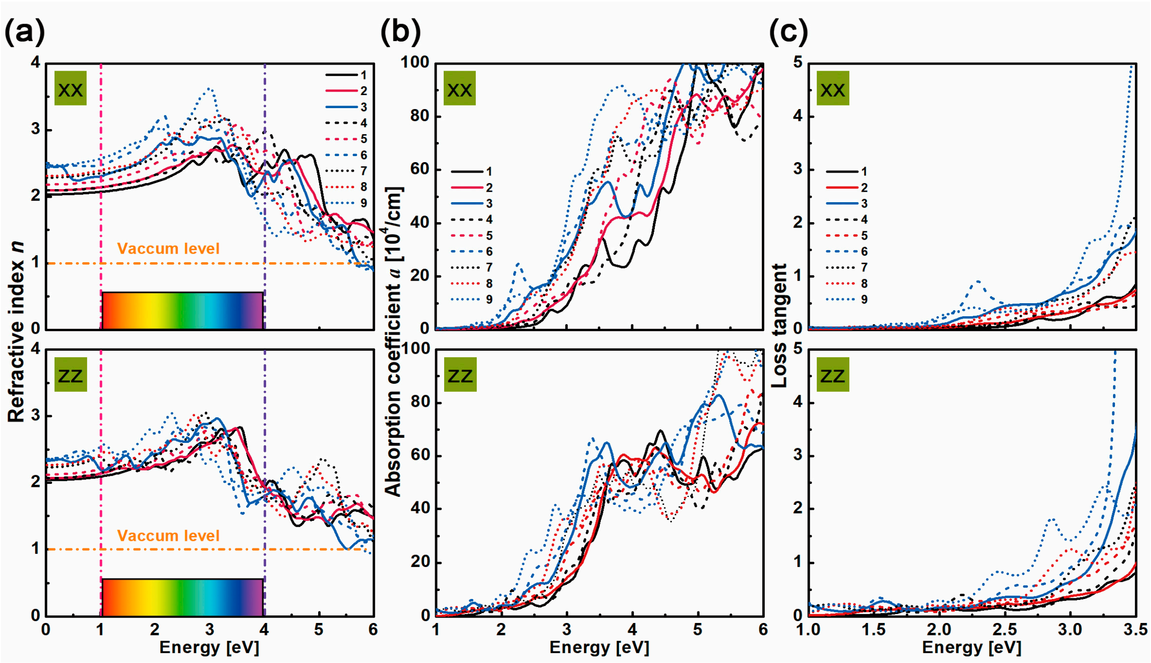

Figure S4. In-plane (*xx*) and out-of-plane (*zz*) refractive indexes **(a)**, absorption coefficients **(b)** and loss tangents **(c)** of N°1–9 GaX/InX.

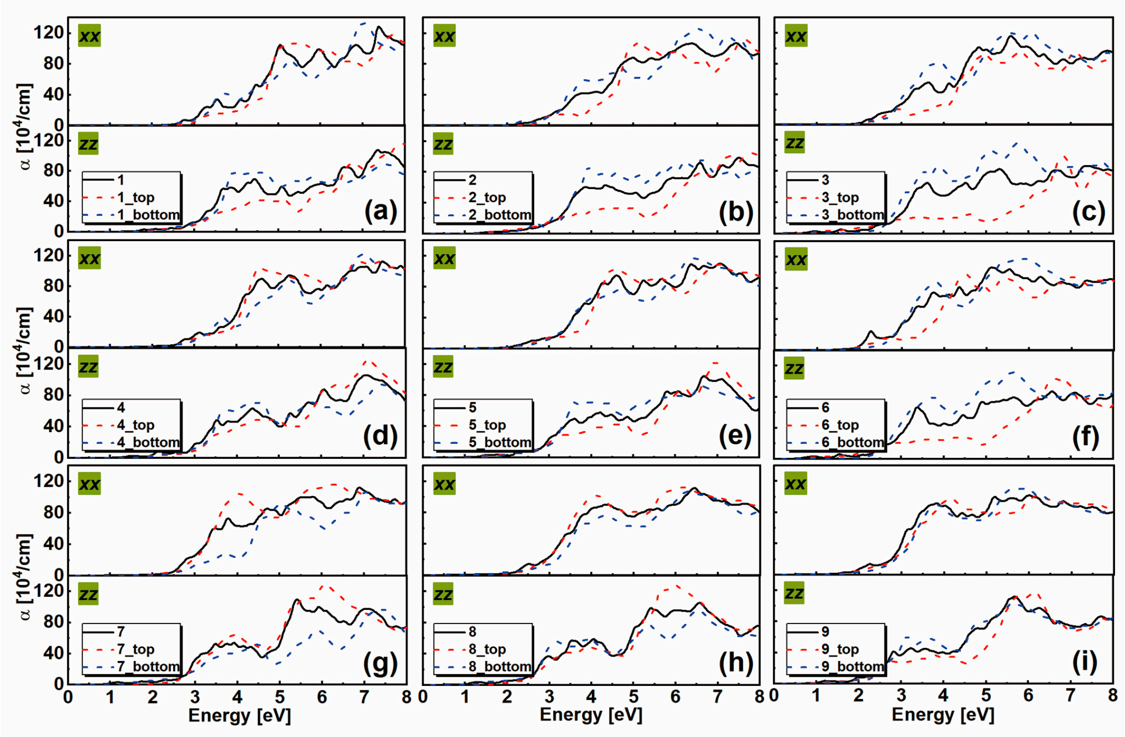

Figure S5. In-plane (*xx*) and out-of-plane (*zz*) absorption coefficients of N°1–9 GaX/InX compared with those of their constitutive top and bottom bilayers **(a)**-**(i)**.

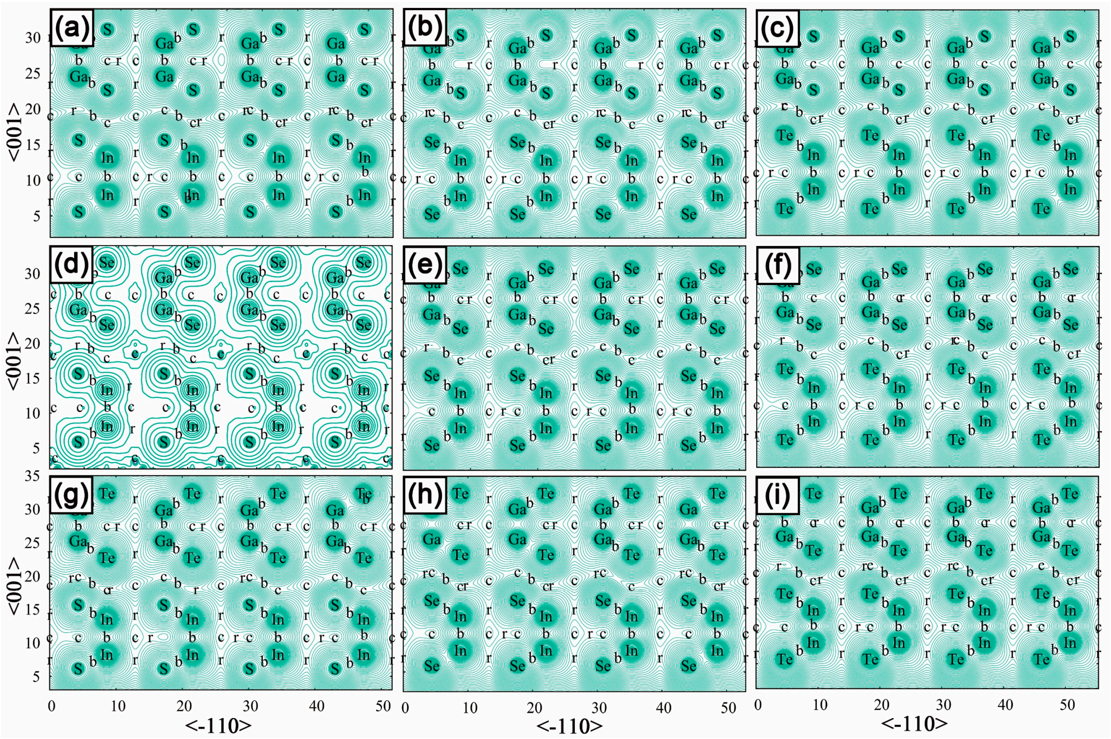

Figure S6. Electron density distributions of N°1–9 GaX/InX along the <110> plane **(a–i)**. Labels "b", "r" and "c" represent bond, ring and cage critical points at zero-flux surface respectively.

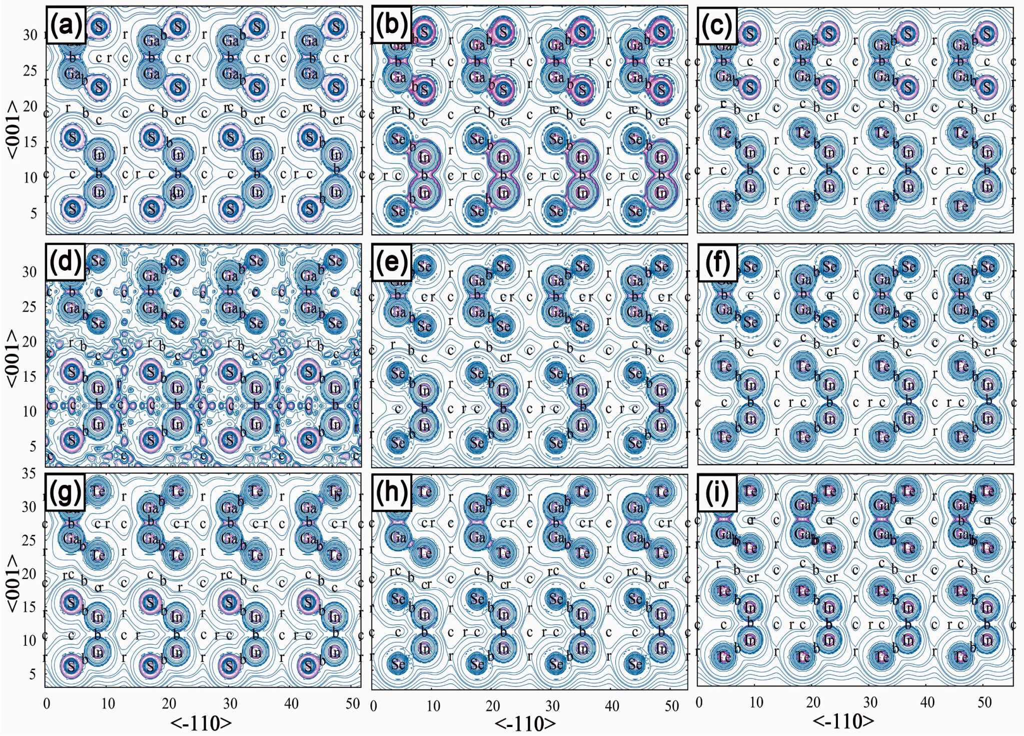

Figure S7. Laplacian of electron density distributions of N°1–9 GaX/InX along the <110> plane **(a–i)**. Labels "*b*", "*r*" and "*c*" represent bond, ring and cage critical points at zero-flux surface respectively.

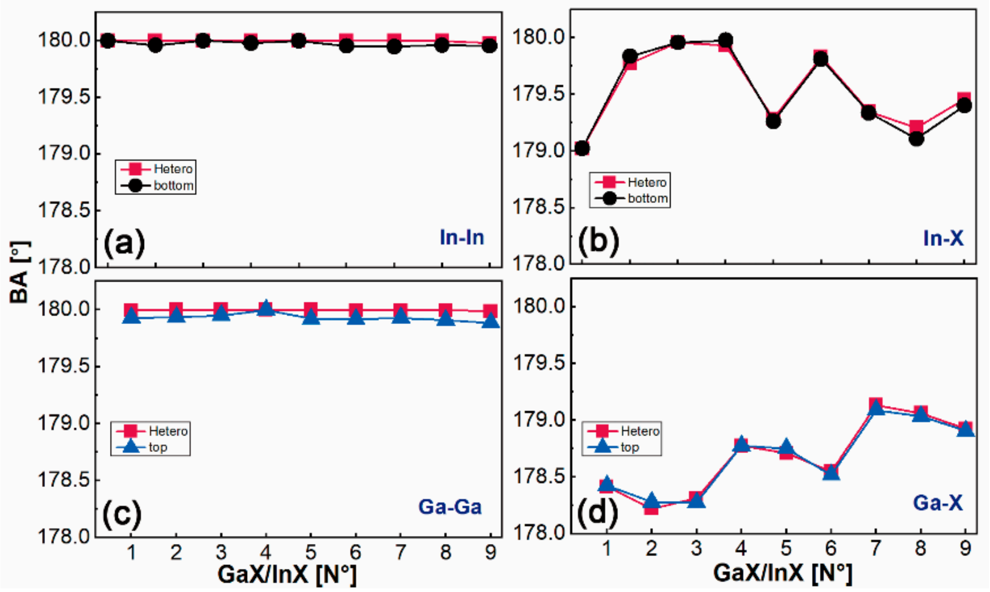

Figure S8. Bond angle (BAs) of In–In **(a)**, In–X **(b)**, Ga–Ga **(c)** and Ga–X **(d)** bonds of N°1–9 GaX/InX (red color), and the constitutive top $[GaX]_2$ (blue color) and bottom $[InX]_2$ (black color).
